# Une amylose AL ganglionnaire pseudotumorale

**DOI:** 10.11604/pamj.2014.19.78.4999

**Published:** 2014-09-25

**Authors:** Faida Ajili, Najeh Bousseta

**Affiliations:** 1 Service de Médecine Interne, Hôpital Militaire de Tunis, Tunisie

**Keywords:** Une amylose, adénopathies, gammaglobulines, amyloidosis, adenopathies, gammaglobulins

## Image en medicine

**Figure 1:**
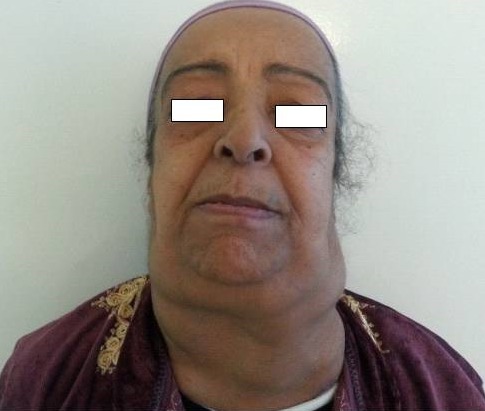
Adénopathies cervicales bilatérales regroupées en magma dont les plus volumineuses mesurent 2 cm de grand axe

Il est exceptionnel qu'une amylose AL se manifeste par à un syndrome ganglionnaire pseudo-tumorale. Il s'agissait d'une patiente âgée de 66 ans, qui consultait pour une voie nasonnée et une obstruction nasale. Elle avait des adénopathies cervicales de 1 à 2 cm de diamètre. La VS, CRP et la NFS était normales. L'EPP avait montré un pic d'allure monoclonale au niveau des gammaglobulines et l'IEPP dans le sang et les urines la présence d'une Ig monoclonale de type IgG associée à une chaine légère Kappa. Le bilan tuberculeux, la PS la BOM étaient normaux. L'IRM des voies aériennes supérieures avait révélé un épaississement de signal hétérogène du cavum et des adénopathies cervicales en magma. La biopsie du cavum et des adénopathies avait montré un dépôt de substance éosinophile acellulaire, rouge brique à la coloration rouge Congo. L’échographie et l'IRM cardiaques étaient sans anomalies ainsi que le bilan rénal. Le scanner n'avait pas montré de viscéromégalies ni d'adénopathies profondes. Le diagnostic d'une amylose AL localisée à la sphère ORL associée à une gammapathie monoclonale a été retenu avec une abstention thérapeutique. Elle présente 2 ans après, une dyspnée d'effort et une aggravation de l'obstruction nasale avec une augmentation de la taille des adénopathies cervicales avec apparition d'adénopathies abdominales au scanner. Un lymphome a été éliminé. Le bilan d'extension de l'amylose était resté négatif. Devant l'aggravation clinique, la patiente a été traité par des cures mensuelles de melphalan et dexaméthasone. Le recul actuel est de 6 mois avec une stabilisation clinique. Figure 1

